# Tricuspid accessory valve–induced hemodynamic deterioration in a Noonan syndrome neonate with myocardial hypertrophy

**DOI:** 10.1016/j.jccase.2026.05.012

**Published:** 2026-06-12

**Authors:** Asuka Miyamae, Keijiro Ibuki, Shinya Takarada, Mako Okabe, Hideyuki Nakaoka, Masaya Aoki, Sayaka Ozawa, Naoki Yoshimura, Keiichi Hirono

**Affiliations:** a Department of Pediatrics, University of Toyama, Toyama, Japan; b Department of Thoracic and Cardiovascular Surgery, University of Toyama, Toyama, Japan

**Keywords:** Accessory tricuspid valve tissue, Right ventricular outflow tract obstruction, Myocardial hypertrophy, Noonan syndrome, Cardiogenic shock

## Abstract

While accessory mitral valves have been reported in association with ventricular outflow obstruction of Noonan syndrome, accessory tricuspid valves remain extremely rare. To the best of our knowledge, this is the first well-documented case report of this unique combination. We report a 2-month-old male infant with myocardial hypertrophy, who presented with progressive right ventricular outflow tract obstruction (RVOTO) caused by accessory tricuspid valve tissue. At referral, echocardiography revealed severe RVOTO due to the prolapse of accessory tricuspid valve tissue during systole. Cardiac catheterization revealed suprasystemic right ventricular pressure. Surgical resection of the accessory tissue led to immediate resolution of the RVOTO. Postoperatively, the patient was successfully managed with beta-blockers and discharged in stable condition. Although the combination of myocardial hypertrophy and accessory tricuspid valve tissue is rare, clinicians should be aware that it may lead to dynamic and potentially life-threatening hemodynamic deterioration due to RVOTO.

**Learning objective:**

In neonates with Noonan syndrome, an accessory tricuspid valve leaflet can cause right ventricular outflow tract obstruction, and early surgical resection can prevent fatal hemodynamic deterioration.

## Introduction

An accessory valve is an abnormal structure associated with the mitral or tricuspid valve, often occurring with other intracardiac malformations [1]. Myocardial hypertrophy in RASopathies, including Noonan syndrome, can cause ventricular outflow tract obstruction. Accessory valves can also affect the circulation; in particular, accessory mitral valves are known to cause left ventricular outflow tract obstruction (LVOTO) [2]. Conversely, studies describing accessory tricuspid valves are extremely rare.

## Case report

**Fig. 1:**
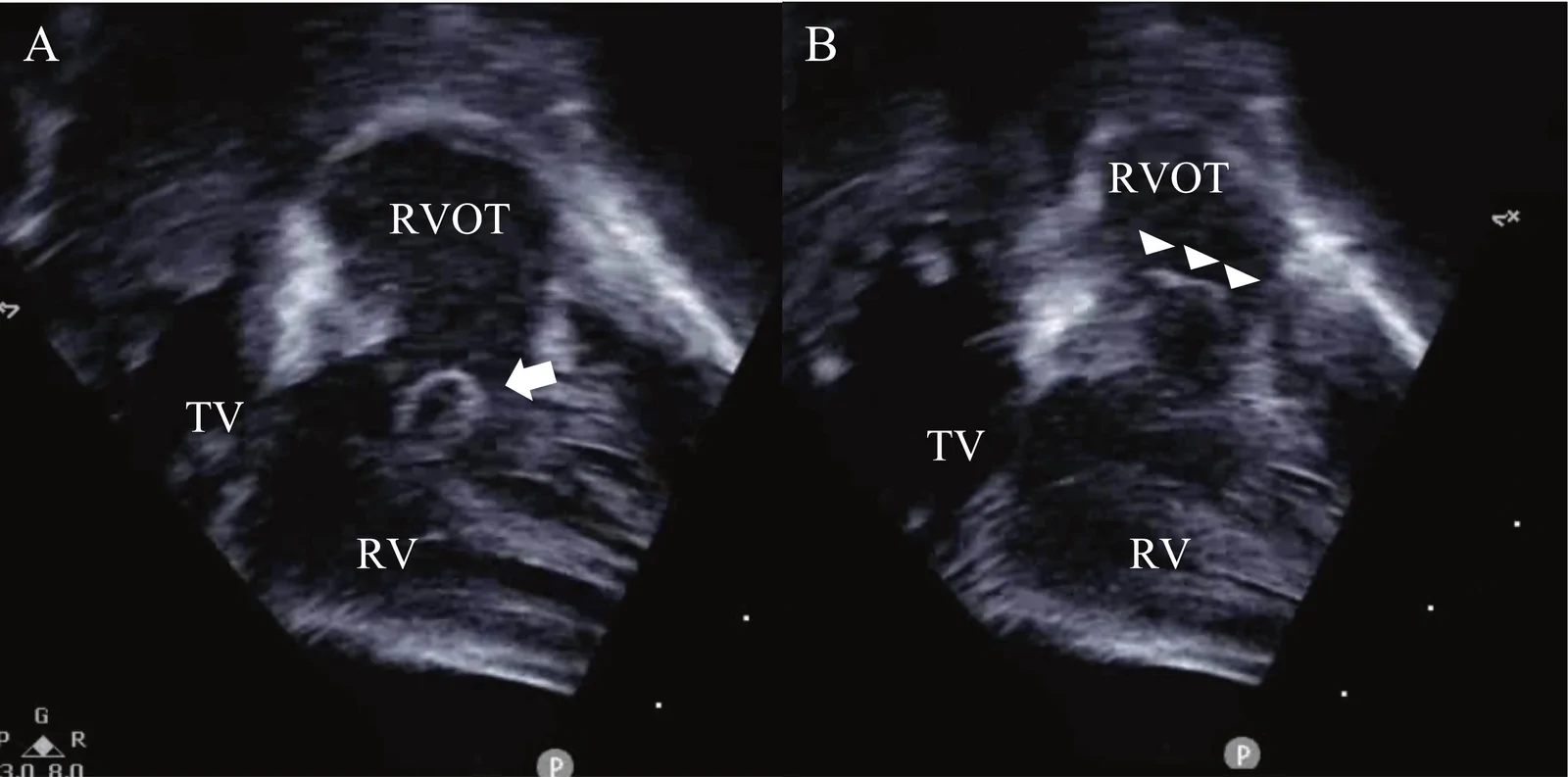
(A) Echocardiographic image of the right ventricle (RV) in the subxiphoid view, showing accessory tricuspid valve (TV) leaflet contiguous with the septal leaflet in diastole (arrow). (B) Accessory TV leaflet prolapsing into the right ventricular outflow tract (RVOT) in systole (arrowhead).

The patient was a male infant born at 38 weeks and 6 days of gestation, with a cardiac murmur noted soon after birth. At the referring hospital, transthoracic echocardiography revealed right ventricular outflow tract obstruction (RVOTO) with a peak velocity of 3.6 m/s, and oral beta-blocker therapy was initiated. At 2 months of age, he was referred to our hospital and admitted for further evaluation. Transthoracic echocardiography on admission demonstrated muscular narrowing of the subpulmonary RVOT, extending over a length of 11.9 mm, with a peak flow velocity of 3.8 m/s and an estimated pressure gradient of 58 mmHg. There were no morphological abnormalities of the pulmonary valve and the tricuspid valve itself showed a normal three-leaflet structure. Both the left and right ventricles showed circumferential myocardial hypertrophy. In addition to the muscular narrowing of the RVOT, accessory valve tissue contiguous with the septal leaflet of the tricuspid valve was observed protruding into the outflow tract during systole. Color Doppler imaging demonstrated marked turbulent flow originating at the level of the accessory leaflet, suggesting that the accessory tissue was the predominant cause of the RVOTO (Fig. 1).

Cardiac catheterization was promptly performed 1 week after referral and revealed a suprasystemic right ventricular pressure of 75/7 mmHg versus a left ventricular pressure of 65/7 mmHg. Right ventriculography demonstrated the accessory tricuspid valve leaflet prolapsing into the RVOT. Following cardiac catheterization, surgical intervention was planned. As his hemodynamic condition was stable at that time, he was temporarily discharged with close follow-up. However, 1 week after discharge, he was readmitted with poor feeding, irritability, and persistent tachycardia, with a heart rate exceeding 180 beats/min (bpm). Intravenous fluids and oral beta-blockers reduced the heart rate to 140 bpm, temporarily stabilizing his condition. Echocardiography revealed increased RVOT velocity to 4.8 m/s, corresponding to a pressure gradient of 92 mmHg, indicating progression of the outflow tract stenosis compared with the values obtained at admission (3.8 m/s, 58 mmHg). Due to impending circulatory collapse, he was admitted to the intensive care unit on the same day and subsequently underwent surgical correction of the tricuspid valve after stabilization.

**Fig. 2:**
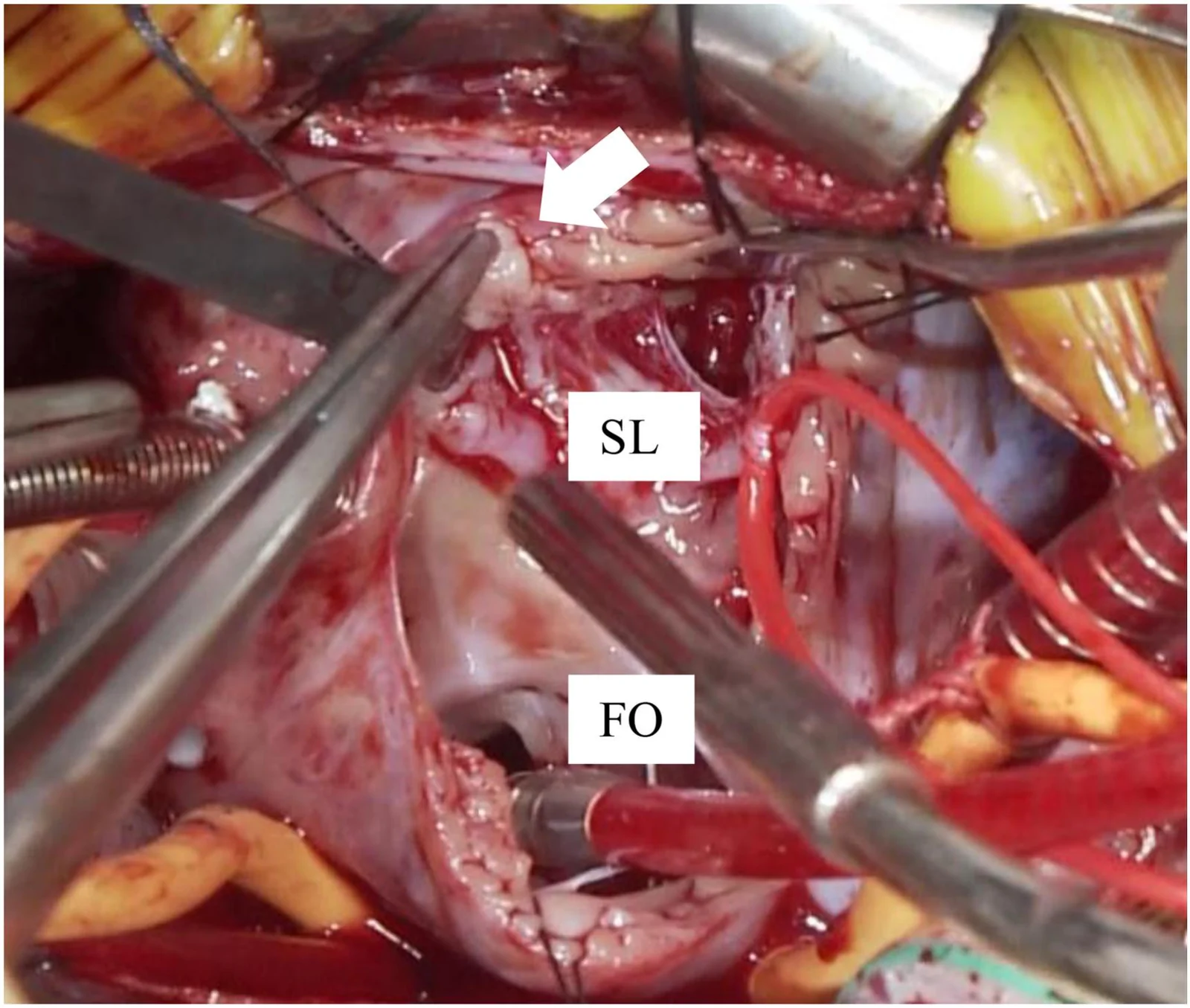
Intraoperative view of the tricuspid valve through a right atriotomy, displaying a redundant accessory tricuspid valve leaflet (arrow). SL, septal leaflet; FO, fossa ovale.

**Fig. 3:**
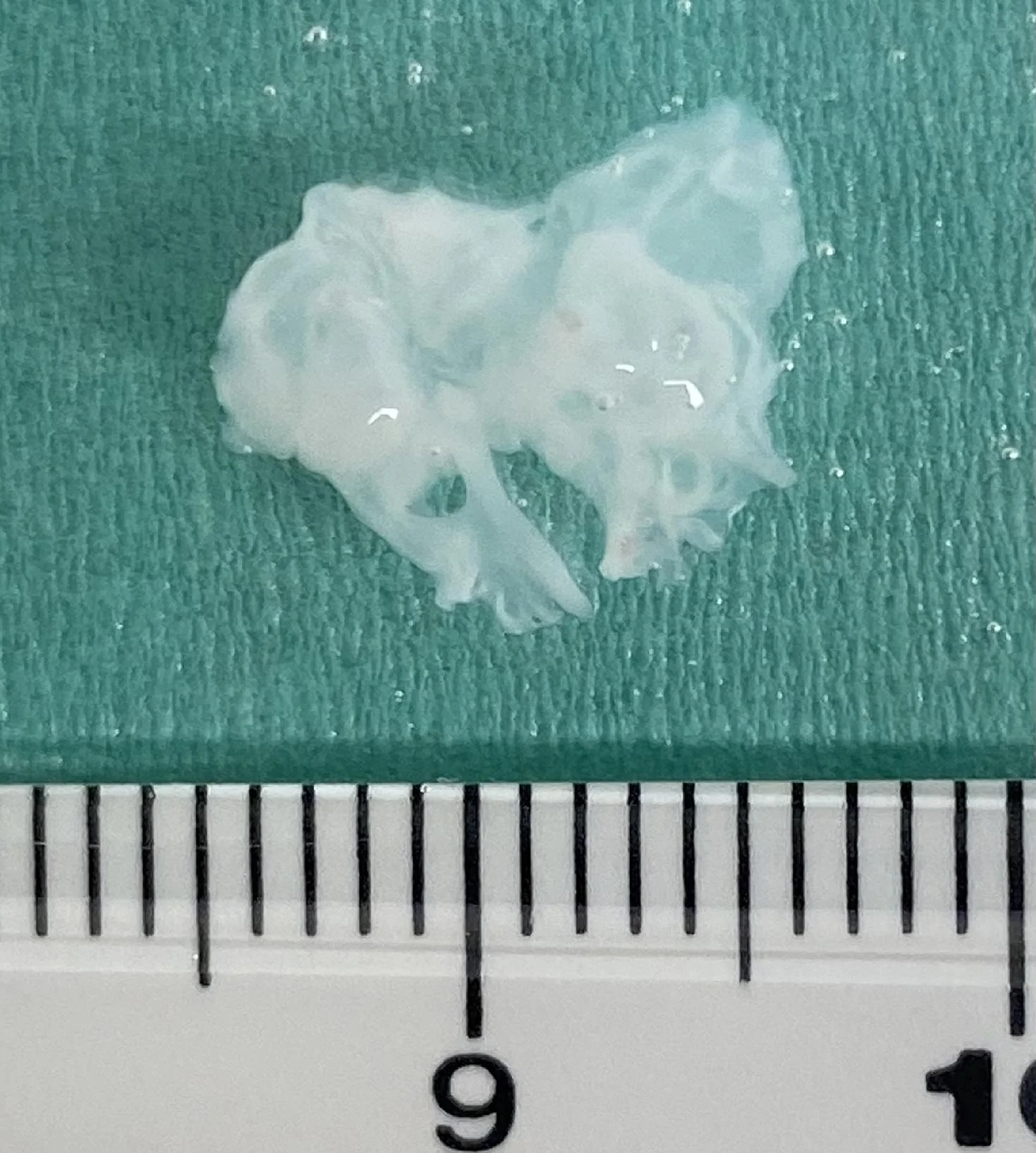
The excised accessory tricuspid valve leaflet.

Intraoperative findings confirmed a long, hypermobile accessory tricuspid valve tissue originating from the septal leaflet, extending into the RVOT and prolapsing beneath the pulmonary valve during systole (Fig. 2). The accessory tissue was carefully resected without injuring the normal chordae tendineae (Fig. 3). After resection, a 9-mm Hegar dilator passed through the RVOT without resistance, and the pulmonary valve showed no stenosis. Postoperative echocardiography confirmed complete resection of the accessory tricuspid valve tissue and improved RVOTO without turbulence, with flow velocity decreasing to 2.0 m/s.

Genetic testing for Noonan syndrome–associated genes did not identify any pathogenic or likely pathogenic variants. Nevertheless, during longitudinal follow-up the patient developed phenotypic features characteristic of Noonan syndrome, including hypertelorism with downslanting palpebral fissures, ptosis, and low-set ears. Taken together with the presence of myocardial hypertrophy, the patient was diagnosed clinically with Noonan syndrome.

## Discussion

We report the first case of acute hemodynamic deterioration due to RVOTO in a patient clinically diagnosed with Noonan syndrome, who presented with myocardial hypertrophy and accessory tricuspid valve tissue. Accessory valve tissue is a rare congenital defect characterized by additional valve tissue attached to normal valves within the cardiac chambers, varying in mobility and attachment, often associated with other intracardiac malformations. Consequently, the clinical presentation of accessory valve tissue ranges from asymptomatic to thromboembolism, heart failure, LVOTO or RVOTO, and severe arrhythmia [3].

The accessory tricuspid valve is mainly linked to congenital heart diseases such as tetralogy of Fallot and ventricular septal defects, with mobility determined by tendon cord length. Longer, more mobile tendon cords may appear as parachute-like structures in the right ventricle or RVOT, occasionally resulting in RVOTO [1].

In contrast, RASopathies such as Noonan syndrome are known to cause various congenital heart diseases due to genetic mutations, with hypertrophic cardiomyopathy and pulmonary valve stenosis being the most common complications. However, approximately 6–7% of cases exhibit mitral valve abnormalities, which can lead to LVOTO [2]. Conversely, reports of tricuspid valve abnormalities in RASopathies are limited. Cases involving accessory tricuspid valve leaflets are even rarer with only a few autopsy cases described, including Ebstein's disease [4].

In this case, accessory valve tissue contiguous to the tricuspid septal leaflet was observed. During systole, the tissue moved forward and prolapsed like a parachute in the outflow tract as observed on transthoracic echocardiography. The immediate postoperative resolution of RVOTO after resection of the accessory tissue alone, without myectomy, strongly suggests that the accessory tricuspid valve tissue was the predominant cause of the obstruction, whereas myocardial hypertrophy likely acted as an aggravating factor by further narrowing the subpulmonary infundibulum. We planned surgical intervention immediately after cardiac catheterization because of the suprasystemic right ventricular pressure and the morphological findings of accessory tricuspid valve tissue. However, despite temporary hemodynamic stability, the patient developed rapid clinical deterioration within a week. This course suggests that patients with dynamic RVOTO caused by hypermobile accessory valve tissue, particularly in the presence of coexisting myocardial hypertrophy, may have highly fragile hemodynamics and can deteriorate unpredictably. Therefore, once progressive Doppler velocity increase or clinical signs such as poor feeding, irritability, or persistent tachycardia are observed, prompt reassessment and early surgical intervention should be strongly considered to prevent circulatory collapse.

## Conclusion

Although rare, the coexistence of Noonan syndrome and an accessory tricuspid valve can rapidly lead to outflow tract stenosis and sudden hemodynamic deterioration, warranting clinician awareness. Early surgical intervention should be considered to prevent fatal circulatory collapse during progressive RVOTO.

## Informed consent statement

Written informed consent was obtained from the parents for the publication of this case report and accompanying images. The Institutional Review Board (IRB) approved the study protocol and publication of data (I2022002).

## Declaration of competing interest

The authors declare no conflicts of interest. This work was supported by a 10.13039/501100001691JSPS Grant-in-Aid for Scientific Research (C) (JP22K08121).
